# Experiences from Decentralised Radiological Services in Norway – a rural case study

**DOI:** 10.1186/s12913-019-4800-z

**Published:** 2019-12-12

**Authors:** Aud Mette Myklebust, Hilde Eide, Brian Ellis, Rona Beattie

**Affiliations:** 1 Department of Optometry, Radiography and Lighting Design, Faculty of Health and Social Sciences University of South-Eastern Norway, Postboks 235, 3603 Kongsberg, Norway; 2 Science Centre Health and Technology, Faculty of Health and Social Sciences, University of South-Eastern Norway, Kongsberg, Norway; 3 School of Health and Life Sciences, Glasgow Caledonian University, Cowcaddens Road, Glasgow G4 0BA, Scotland, UK; 4 Glasgow School for Business and Society, Glasgow Caledonian University, Cowcaddens Road,Glasgow G4 0BA, Scotland, UK

**Keywords:** Case study, Decentralised radiological service, Organisation, Quality, Cooperation, Funding

## Abstract

**Background:**

Implementation of the Norwegian government’s Coordination Reform (2012) aims to decentralise health care services from centralised hospitals to local communities. Radiological services in Norway are mainly organised in hospitals, because of the significant financial and human resource demands engendered by the need for advanced technological equipment, and specialised staff. Some selected conventional x-ray services have been decentralised into rural communities. The purpose of this single case study was to highlight experiences from different stakeholders’ of organising decentralised radiological services in a rural area in Norway.

**Methods:**

A qualitative single case study design was adopted, collected data using focus groups with healthcare professionals and managers to obtain stakeholder’s experiences of the radiological services in this rural area. The key emergent themes from the literature, decentralisation, quality, professional roles, organisation and economic consequences were discussed with each focus group. Thematic analysis was used for analyzing the primary data collected.

**Results:**

Four main themes emerged from the focus groups: 1) organisation, 2) quality and safety, 3) funding of radiological services and 4) cooperation between health care professions and health care levels. It was found that the organisation of decentralised radiological services to rural areas is challenging because of the way health services are structured in Norway. The quality of service was found to be inadequate in some areas because of the superficial level of training given to non-radiographic staff. The experience is that the Norwegian funding system hinders an efficient decentralised health care service. Effective cooperation and responsibility between health care professions and levels was challenging. There needs to be improved co-working by clearly defining roles and responsibilities.

**Conclusions:**

A key recommendation for the organisation of rural radiological service was the development of a satellite link with an acute hospital. Quality of the service could be improved and should be given priority. Structural change to the financial system whereby money follows patients, might also facilitate more patientcentred services across healthcare levels. Improved mutual understanding between rural radiological services and hospital specialists and managers is important for a high quality and consistent radiological service to be delivered across Norway.

## Introduction

Over the past decade, there has been a tendency worldwide to decentralise health care services [1]. Governmental initiatives and a number of reforms have been implemented in many countries [1–3].

Decentralisation can be defined as the transfer of authority and responsibility from central government to the local level, with the aim to promote local autonomy [4, 5]. Decentralisation builds on the idea that smaller organisations are more responsive and accountable regarding health services to the public than larger organisations [4]. Saltman and Bankauskaite [6] define decentralisation in terms of three key functional dimensions: political, administrative and fiscal. Political decentralisation has traditionally been referred to shifting policy making responsibility from a centralised system to several local ones [7]. Administrative decentralisation is about developing an effective service, building an infrastructure and developing a workforce policy. The size and composition of the workforce is critical for a high quality service [8]. Rondinelli [5] also defines decentralisation as a four-part framework of devolution, decentralising, delegation and privatisation. The effects of decentralisation have been difficult to estimate owing to its complexity and therefore making it challenging to gather relevant systematic data over time [9]. However, Saltman, Bankauskaite & Vrangbæk [4] argue that decentralisation might have positive effects on healthcare: 1) limits costs due to local decision-makers having better knowledge of the needs and the supply system compared to national bodies, 2) easier for a local government to meet needs and services and to ensure interventions are useful, 3) local communities and citizens benefit from greater participation in building their own healthcare system and 4) the challenge of the ageing population and a concomitant increase in chronic diseases; the integration of social care and healthcare services locally make it easier to respond to these needs [4]. Other advantages include a more coherent and unified health service, the improved implementation of health policy, greater equality in services between rural and urban areas, lower cost containment, community financing and improved intersectional coordination [10].

The purpose of changing from central to decentralised health services is to offer an improved health service to the population, including better quality, greater efficiency and lower costs. Despite this, it seems like each country has its own economic, political and historical reasons for decentralisation, and therefore also have developed the health care system differently [11]. Experiences from Spain, Italy and Scandinavian countries is that efficiency, though not automatically, will be improved. A clear definition of different stakeholder’s roles and manager responsibilities seems to be of importance [11]. Human capital is one essential challenging component to consider when implementing decentralised health care. The need to invest in human capital mostly doctors, to cope with the ever increasing demands on healthcare services, particularly in the developed world, has been recognised [12]. Human capital investment has been supported through a global recruitment drive and investment in radiology training particularly for radiologist who are a key specialty and recognised as a scarce medical profession in countries such as Scotland which faces similar demographic and geographical challenges as Norway, [13]. Another challenge is the electronic sharing of medical imaging [14].

In Norway there has been a recent growth in decentralised health care [15], which creates demands for human capital, both in terms of capacity and capability, with quality and staff/patient safety vital attributes of the latter. This increase is in part explained by the Coordination Reform (2012), creating a vision for the future organisation and delivery of health services in Norway. The aim of the Coordination Reform was to treat people closer to home, to decentralise specialised health services to a larger extent, and to shorten the length of inpatient treatment in hospital [16]. During the implementation of this reform, a number of health care services moved from being hospital based to providing local based treatment, including some radiological services. Radiological services are still most frequently delivered in large city centre hospitals.

It is widely accepted that there is a demographic trend worldwide towards an increasingly ageing population. This situation will require more complex health care services and increase in health care costs. To reduce costs and make health care services more easily accessible there is a trend towards decentralising health care services. Challenges in the interaction between the different levels within the Norwegian healthcare system, for example, regarding different funding systems and uncoordinated computer systems, have been recognised in the coordination reform [16].

The population of several counties in Norway is dispersed over large areas. The decentralisation of medical services through an expansion of the scope of the primary health care sector may offer solutions to the current problems by enabling the delivery of health care services to rural areas at the point of patient need through the deployment and development of appropriate staff.

According to the Norwegian Directorate of Health [17], 216 of total 422 municipalities are defined as rural. These municipalities are located at a distance of more than 50 km from a hospital with acute services.

To date international literature about decentralised radiological service is limited and there has been little advice and research globally to inform healthcare professionals about how to establish radiological services in rural health centres [18].

For example a study from New Zealand about community based use of radiological services found that Chest x-rays were the most requested investigation [19]. Whilst an Australian study indicated the importance of training for rural non-radiographic x-ray operators as well as the need to participate in a radiographic network to get feedback on image quality and protocols. The authors stressed that a radiologist should interpret the images [20]. Other studies pinpoint the need for e-learning and management support [21] and the difficulties raised by transferring radiographic tasks to other non-radiographer professionals, such as physicians or nurses [22]. A Canadian study for rural radiotherapy services suggests that the technology to maintain technical quality at remote locations is available, however the big challenge is the maintenance of professional competence in limited staff institutions [23]. Given the scarcity of literature to address decentralisation of radiological services, this study endeavours to add knowledge by contributing to our understanding of the challenges posed by the change of implementing radiological services at a local level, based on the experience and perceptions of stakeholders and the consequent findings will be of importance for decision makers at the central government level (macro level), managers in rural areas (meso level) and in hospitals as well as staff and patients (micro level). **The objective** of this study is to highlight essential experiences and recommendations from resource persons in a rural decentralised radiological service in Norway.

### Organisation of decentralised health care in Norway

Challenges in the interaction between the different levels within the Norwegian healthcare system have been recognised for many decades [16].

Norway has a population of 5 million people and there are three independent government levels; the national government, the counties and the municipalities. Public health care services in Norway are organised on three levels: national, regional and local as shown in Table 1 [24]. At a national level the Ministry of Health and Care Services has overall responsibility for policy decisions, health care financing and the setting of priorities for health care services. Delegated responsibility is given to the regional level. It is the regional authorities who run the hospitals and have responsibility for specialist health care. The focus of this secondary health care system is on specialised hospitals, and the treatment and management of both somatic and mental conditions. The hospitals are organised in four large health-regions, each of which is responsible for specialised health care. In the case of some less common diseases, for example rare cancers, services are concentrated in one national centre of excellence. At the local level, the municipalities are responsible for a range of primary care activities including health promotion, the GPs, emergency units, home care, nursing homes, children and mother care, and some aspects of mental health care.

**Table 1:** Hierarchy of the health care levels of control in Norwegian public health care

National level Ministry of Health Care Services	
Regional level	
Four health regions South – Eastern Norway Regional Health Authority Northern Norway Regional Health Authority Central Norway Regional Health Authority Western Norway Regional Health Authority	Four health regions provides services in 16 counties
Local level 434 municipalities	

In Norway, decentralised radiological services are organised in different ways; some are decentralised and funded by a regional hospital, while others have been established by a municipality from which they also receive funding. These differences in organising depend on what each municipality’s own local government decides. There is a scarcity of research as to the effectiveness of implementation, their organisational structure and effectiveness, and stakeholder satisfaction in implementation of decentralised radiological services.

Differences in use rates of radiology services between urban and rural areas in Norway has been observed [25], but these differences were not quantified, nor has there been any measure of quality. The trend indicated that rates per thousand of examinations were higher in urban areas compared with rural areas.

### The study context

One rural area in Norway was chosen as a case for this study; this area has a three-fold radiological service: 1) an LMC, 2) a regional hospital and 3) six local GP practices.

The LMC, was established in 1979 and therefore had many years of relevant experience in the provision of health care service in a rural area. The radiological service was established in 2002 as a satellite from the regional hospital, with one conventional x-ray machine for skeletal and lung imaging. This unit was operated by a radiographer. All images was digital and sent to the regional hospital to be interpret by a radiologist. The LMC serves six municipalities in the area with approximately 20,000 inhabitants, and is a decentralised community hospital with 14 beds, located 170 km away from the host hospital and offers acute and emergency services, several kinds of telemedicine supported by specialist consultants, midwifery services, dialysis, and palliative cancer care, they also offers radiological services five days a week (Monday to Friday) between 8.00 a.m. and 4.00 p.m. The areas regional hospital offer all conventional x-ray examinations, and also CT and MRI examinations. The x-ray service at the hospital are operate the whole day.

In the district surrounding the LMC 26 GP’s are practising. The number of GPs in this region is sufficient compared to other regions in Norway [26]. Six of the GP practices had their own x-ray machine, operated by non-radiographic staff.

## Method

Given the three-fold radiological service as earlier described and a long experience from decentralised health care services in this rural area a qualitative approach was adopted to reflect different profession’s experiences, perceptions, opinions and challenges of stakeholders working in, and using, decentralised radiological services The design of the study was explorative and draws on a single embedded case study [27–29]. The choice of method for data collection was focus groups in an attempt to get key persons working with decentralised radiological services into discussion and to encourage them to share their experiences. Representative stakeholders were invited from the whole region. The rationale for the selection of participants from the LMC and from the six municipalities in the region, and also from the regional hospital was to get different profession’s perspective from managers and staff involved in the service.

### Participants

The inclusion criteria was; key personnel with significant experience working with decentralised health services, in the LMC, municipalities and in the regional hospital. When the invitations to potential focus group participants were sent out, a letter outlining the study and its aim was attached to the e-mail. The invitations were sent out three weeks before the planned date of the focus group. The timetable for the meeting was not feasible for all the participants who wanted to take part, and it was therefore modified to suit the majority. Arrangements and clarifications for the focus groups were made by e-mail. The information letter included a consent form which was also sent by e-mail. These forms were also printed out and collected at the interview location.

Data collection was organised in two focus groups. The decision to group the participants in this way was taken partly based on the researcher group, and partly as a result of consultation with people in the field. According to Halkier [30], focus groups should not be too homogenous. In the two focus groups in this study there was a mix of professions and positions: physicians, radiographers and managers in different roles. Overview of the focus group participants are shown in Table 2.

**Table 2:** Overview of the focus group participants

Participants Focus group1	Participants Focus group 2
Head Physician – GP manager Participant 1	Head of Radiological unit (radiographer) Participant 7
Radiographer Participant 2	Head of medical services (Radiologist) Participant 8
District Medical manager 2 Participant 3	Head of IT services (radiographer) Participant 9
District Medical manager 1 Participant 4	Coordination manager (physician) Participant 10
Head physician LMC Participant 5	
GP Participant 6	

All participants had from 15 to 25 years of work experience. Participants in Focus group 1 had a wide geographical spread and participants were representative of the entire rural area. Focus group 2 consisted of four participants from the regional hospital, these were all key personnel with 15–20 years of experience.

Focus group interviews was carried out by AMM and HE.

### Data collection

A semi-structured guide (Additional file 1: Table S1), developed by AMM based on an earlier questionnaire amongst the 26 GPs in the region. Quality in service, organisation of the service, funding and the professional role of in decentralised health care were discussed. The choice of a semi–structured guide enabled the participants to share their experience and views [31]. The focus groups were conducted over a 2 month period, moderated by the first author (AMM) and supported by the second author (HE). The focus groups were recorded, focus group 1 lasted 60 min and was carried out at the LMC. Focus group 2 lasted 52 min and was carried out at the regional hospital. A report from the focus group was provided and sent to the participants for comments, all comments was taken into account.

The Norwegian Centre for Research Data approved the handling and storage of personal information in this study. The Norwegian Centre for Research Data considered approval of this study by the Ethical Committee to be unnecessary.

### Data analysis

Qualitative data from the focus groups were analysed using qualitative thematic analysis [32, 33]. This thematic analysis used four stages as can be seen in Table 3 below, where the first stage was familiarising with data. The data from focus groups was transcribed by AMM shortly after they took place and then reading and re-reading of the transcribed material was undertaken and initial ideas noted. The second stage was to highlight relevant material and define descriptive codes. The third stage of analysing the data was clustering descriptive codes and interprets the meaning of them. Stage four was to define and name themes. The data analysis was mainly carried out by AMM and discussed in the team. These discussions around the analysis increased the trustworthiness. However, bias cannot be ruled out, but it can be minimised by using King and Horrocks [33] procedure (Table 3) and by more than one person analysing the data or by discussions among researchers involved in the study.

**Table 3:**
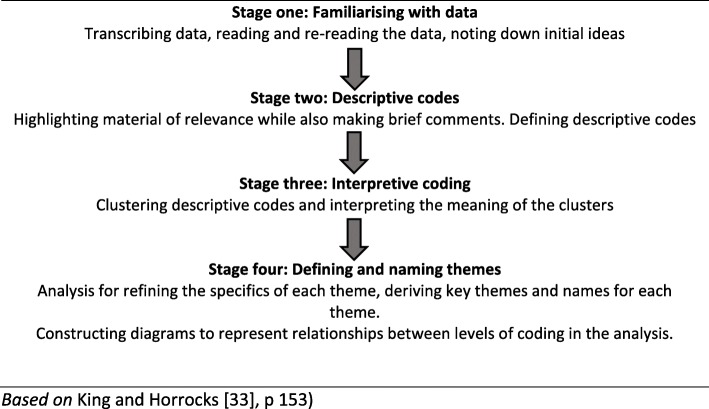
Stages in thematic analysis

*Based on* King and Horrocks [33], p 153).

## Results

Four themes emerged from the thematic analysis: organisation; quality of the service; funding and cooperation. Each will be discussed below. In addition there was support for the principle of decentralised services, which is discussed next.

### Decentralisation

Participants in the two focus groups were satisfied with the x-ray service at the LMC, and agreed on the need for a decentralised radiological service given the long distance involved in travelling to a hospital (a drive of about 3 to 4 h). One GP said:

*“The municipality managers and the GPs want a good health care service, a local service …*.”

Political policies around decentralisation were highlighted, such as the Coordination Reform, and in particular focused on patients’ rights to equality in health services that should not depend on whether patients live in a city or in rural areas. A radiologist stated:

*They (the patients) have the right to exactly the same health care as someone who lives in a central area, so then it’s not really about money, we have a duty that we must fulfil, and this is what we must try to achieve.*

### Organisation

The health care and the radiological service was organised as a three-part service with: 1) x-ray facilities at the regional hospital, 2) x-ray facilities at the LMC, and 3) x-ray facilities at the GP’s practices or at the emergency unit, placed within the LMC.

All the participants agreed that they were very satisfied with this three-fold organisational model and described it as unique and special. One district manager said:

*“I think that as a region we are a bit special, given that every GPpractice has x-ray equipment; that is not common in Norway … …*

Another district manager remarked that:

*“We have unique arrangements of x-ray service in our region”.*

### Quality and safety of the service

The participants volunteered points related to the quality of the service, without the moderator’s intervention, thus suggesting its importance as can be seen by participants agreeing that they were offering a high quality health service. A GP manager said:

*“High quality health care services that make our region a good place to live in and good for tourists too”.*

Nevertheless, three topics emerged regarding quality: radiation protection, training of staff at GP practices about positioning and technical x-ray issues, and resources. Despite the view that they were offering a high quality service, GPs also admitted that image quality could sometimes be better. The mobile x-ray equipment at the GP practices was limited and inappropriate for the examination of, for example, hips and spine. A GP reported that:

*“We have seen that that the images may not be good enough as we have noted in feedback from the orthopaedic surgeon”*.

The participants from the hospital were clear about the need for high quality of the images taken by GPs to save patients from unnecessarily long trips to the hospital and possible inappropriate treatment. The quality of images sent from the GPs practices, was of therefore a concern. No radiographer was available in the GPs practices, therefore a nurse, medical secretary or the GP was performing the imaging. A radiologist said:

*“The principle is that it is not a radiographer taking the images up there* (in the rural area)*, these people are basically amateurs and the radiologists have been rather concerned about the quality of the images and the projections”.*

They stated that training of staff in the GP’s surgery is important and a discussion of training needs focused on positioning, technical aspects and radiation protection. The understanding of the radiographic principle ALARA (As Low as Reasonably Achievable) seems, alarmingly, not to be known by staff working in GP practices, and is clearly an example of a human capital capability need that requires urgent attention.

One of the solutions, which is challenging for the “model” of organising the radiological services in the studied area is legalisation regarding radiation equipment and protection. Norwegian legislation distinguishes between installed fixed x-ray equipment (x-ray machine that is installed permanently in one location) and simpler x-ray equipment (x-ray machine that can be moved from one location to another).

When the GPs take x-rays and interpret the images themselves in certain situations a second opinion may be required to make an accurate diagnosis for the patient. The focus group participants described technical limitations in their communication with the hospital. The images were sent by e-mail, or smartphone, and image quality was therefore often reduced for this reason. In particular, it was noted that the orthopaedic surgeon was unable to interpret inferior quality images prior to their transfer to the hospital and:

A head physician said:

*“...Often the orthopaedic specialist does not draw a conclusion based on the images taken by GPs”.*

To solve this problem the GPs participating in the focus group wanted a system that might allow digital images to be sent directly to the hospital imaging system for second opinion. However, this could potentially create further concerns regarding data security, as appropriate digital security protocols would require to be observed including encryption and password protected. Whilst, still a relatively low risk recent research, conducted since this study’s fieldwork, by Ben Gurion University in Israel [34] highlights the potential risk that radiological equipment, as part of the wider Internet of Things (IoT) could be vulnerable to corruption by cyber criminals or terrorists. This suggests a further training need for all staff involved in the use of radiological equipment and materials on top of what is already recommended by the International Atom Energy Agency (IAEA) [35].

### Funding

The focus group participants stated and agreed that decentralised radiological services save society a lot of money. Saving patients long travelling distances and ambulance capacity are expected to save money, but also travelling time for patients and relatives [36]. This economic saving was emphasised by a physicist:

*“For society – and the health economy we win on this. Also for our ambulance capacity having a local x-ray service means that ambulances can stay in region. An ambulance trip to the hospital costs about £2,000. It is expected to save about 2,000-2,500 ambulance trips by having x-rays in the GPspractices”.*

The total saving for society will based on this quote be at minimum *£* 4,000,000 each year. Thus investment in human capital can bring socio-economic returns.

### Cooperation

A main theme that emerged was cooperation between the hospital and GPs. The participants raised the matter of importance of the need for good cooperation with the hospital to provide the best possible services for patients. The participants experienced very good cooperation between the hospital and the GPs in the region. Indeed one district medical manager in the region highlighted:

*“In our experience the hospital is genuinely concerned with good cooperation in our district so making a good decentralised healthcare is a common goal”*.

However, participants in the hospital focus group raised concerns about the distribution of responsibility between the hospital service and the GP imaging service, and they were clear about the fact that the hospital was not involved, and should not be involved, in GPs’ imaging practice. A head of IT services said:

*“It is quite clear that it’s the primary health service that takes the image and is responsible for the patient until the patient is referred. It’s only when the orthopaedic surgeon says “you need to refer the patient“ that the hospital assumes responsibility, and; until that point,it’s the primary health service’s responsibility”*.

The hospital participants were also sceptical about the principle of the current practice where GPs can obtain x-ray equipment. Despite this however, the hospital wanted to improve the quality of the GP imaging service by developing a system where the hospital IT system could receive images and orthopaedic surgeons could access the images and provide a second opinion. The head of medical services at the regional hospital said:

*“The cooperation could consist of the images being sent to us (the hospital) by local doctors. We could consider them before patients are treated. Otherwise local doctors take the images and deal with them (the images) themselves”*.

To summarise the findings from the focus groups, the participants highlighted the need for decentralised radiological services. Particularly, as they felt this service was beneficial to the patients and saved society a lot of money, particularly as far as the ambulance service was concerned, and also that the travelling time for patients was reduced.

Participants from the district were very satisfied with the existing three-part organisation with a radiological service being provided in GP practices, at the emergency unit in the LMC and at the hospital. In contrast, participants from the hospital were worried about the quality of the images and the radiologic competence of staff at the GP surgeries. They would like to establish a new service for the GPs with the possibility of sending x-rays to the hospital IT systems for a second opinion.

**Fig. 1:**
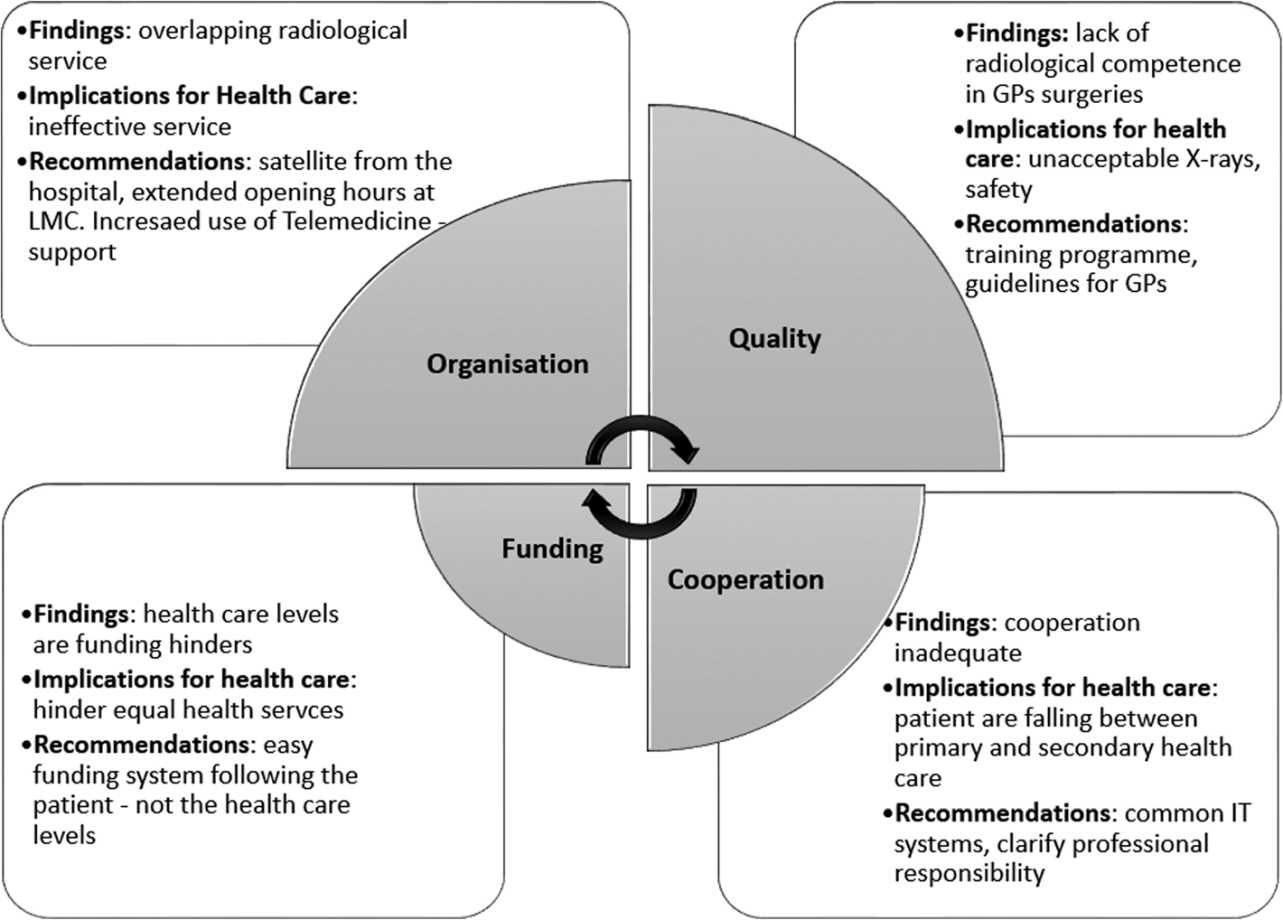
Key findings, interaction, relative weighting implications for decentralised health care and recommendation

GP’s themselves acknowledged that image quality was sometimes poor and a lack of radiological competence in the GP practices since GPs, nurses or even at times a medical secretary carried out the radiological imaging, which would be highly unusual in other developed countries’ health systems. Key findings are illustrated in Fig. 1.

## Discussion

**The objective** of this study was to highlight essential experiences and recommendations from resource persons in a rural decentralised radiological service in Norway.

As this research has shown; decentralised rural radiological services face several challenges regarding organisation, quality and safety, funding and cooperation. The main findings are summarised in Fig. 1 and each area will be further discussed.

### Organisation of decentralised radiological services

As described in an earlier study by Pavoloni and Vicarelli [37], one of the benefits of decentralisation is that this brings the health care services closer to where people live and is confirmed in this study. Saltman and Bankauskaite [6] have identified different models for organising decentralised health care. As previously mentioned the rural radiological service in the studied district is organised in three-fold. However, these three layers of provision of radiological service found in this rural area have different ‘owners’. A consequence of these different health care levels and ownership is that decision-making processes in these organisations are not integrated. The radiological service at the LMC is a satellite from the regional hospital, and decisions follow the hospital’s protocols. Whereas decisions for the x-ray service in GPs practices is taken by each GP in collaboration with the health system in each municipality. For the third service, in the acute unit, decisions are taken by the service itself in collaboration with municipality coordination. This is complex and may lead to local systems or “personal decisions” made on a more ad hoc basis, rather than a coherent service. Consequently, there may be a risk that health care services have different service provision and different standards of service in different areas.

With the current organisation, there appears to be an overlap in radiological services in this region, and there is probably potential for improvement in the organisation, which would result in a more cost-effective service, possibly of higher quality. Recommendation for a high quality service is to have radiological service as a satellite from a hospital, were radiologic competence is available. In addition increased use of telemedicine support. As Roberts et al. [23] states, the technology now exists to maintain technical quality at remote locations.

### Quality in decentralised radiological services

Quality can be defined by factors such as efficiency of services, safety, the possibility of user interaction, coordinated services, the efficient use of resources, availability of services and their equal distribution [38]. This is a challenge in rural areas. The quality indicators at stake in this study were mostly related to no radiography staff at the GPs practices as well unsafe use of x-rays. Hana and Rudebeck [39] states that the professionals working in rural areas play an essential role for quality of health care services when these services are decentralised. Participants in the focus groups confirmed the need for experienced, dedicated high-quality staff, and reported a high level of satisfaction with the radiographer working at the LMC, however they could only provide limited coverage of the area and time. Overall there were limitation in human capital, both in terms of capacity and capability. To address this gap dedicated and competent staff are essential in a decentralised health service, and the competence of administrative staff and those in leadership positions in primary care are also of importance for the quality of health services in rural areas. This is in line with studies from Australia and New Zealand [19, 20]. The people working at the GPs practices (physician, nurse and secretaries) as well as at the LMC and the regional hospital were all very committed to deliver high quality services, however that does not mean they have radiographic competence. The recommendation is that non-radiographic staff need competence and training in x-ray imaging.

The issues mentioned above may lead to discussions amongst professionals about skills and responsibility. Different professions may wish to protect their own discipline, without reflecting on possible consequences for patients. This inter-disciplinary rivalry presents a challenge for professional identity, for example image quality, radiation protection and standards are an inextricable part of the radiographer’s professional role, and in addition to the radiologists’ role in diagnosis and treatment. In contrast, the GP’s role is to diagnose and treat, and they perceived image quality and radiation dose as relatively minor concerns [22]. There is therefore an urgent need for continuing professional education and a closer connection with a radiological environment for both radiographers and other staff who perform x-ray imaging in rural areas, a point also highlighted by Smith and Jones [22].

Smaller operating units will probably offer poorer quality than larger ones, partly because qualified staff in all disciplines is more difficult to recruit and retain. Health care services in rural areas are made up of small organisations, are distributed across extensive areas, lack qualified staff and a large range of tasks have to be performed by a limited number of staff [39].

Fredriksson and Winblad [1] found in their study that a decentralised model for health care services results in inconsistency in rules and regulations. In this study it was found that there is a lack of clear and consistent x-ray procedures in GP practices, and decision-making based on x-rays or clinical examinations differed between GPs, depending on their experience, which was also found by another study identifying GP- related factors such as experience, as an important factor for rural practices [40]. Indeed in the region studies there were differences in the working experience amongst GPs which influenced their refferal practice to specialised health care; experienced GPs reffered less than not experienced GPs. Participants in the focus groups confirmed the need for experienced, dedicated high-quality staff, and reported a high level of satisfaction with the sole radiographer working at the LMC.

When discussing quality and staff competence, radiation protection is an additional issue. One of the provisions of the Regulations on Radiation Protection is the presence of a responsible doctor. The regulations state that x-ray and MRI machines have to be operated by radiographers or a doctor with a relevant specialty [41]. Personnel using x-ray equipment should undertake training in radiation protection and use of radiation related to each working tasks every year. Clearly, this is not the case in the GP practices.

### Funding

According to Saltman and Bankauskaite [6] decentralisation may lead to lower costs, since lower planning and administration costs are closely related to political decentralisation. Local politicians can have greater influence on how to use money in their own area. There is however a lack of consensus regarding the benefits of economic decentralisation. Dilemmas such as how to use taxes payment [42] and disagreement about policy for financial grants from the central government to local level governments are some of the issues still to be resolved.

Duplication of services in decentralised health care as is found in this study may make these services more expensive than central government controlled services. Results from this study indicate that the region studied has the potential to achieve a more economically efficient radiological service, due to the current organisation being three-part and, in some areas, overlapping. A key issue is the balance between effectiveness and efficiency. The fact that the Norwegian health system illustrates in Table 1, are organised in different levels, also have impact to the funding.

### Cooperation

The way health care services are organised in Norway seems to hinder the efficient organisation of decentralised health care services. To provide high quality health care services to patients in all situations, the cooperation between the health care levels, primary health care and specialised health care, must be raised to a higher standard than is currently the case. The coordination reform [16] aimed to reduce these problems, and health care managers are working to reorganise the services by improving and increasing cooperation between levels.

Regarding the Cooperation reform [16] there is a need for cooperation between the levels in health care, with the aim of providing better health care for the patients. Participants in focus group one, felt that the cooperation reform had contributed to improved cooperation between the rural area and the hospital. The reform had also improved the hospital’s understanding of the rural area’s needs. Because of the systematic cooperation, the hospital’s thinking and understanding of needs in the rural area was increased, and has already resulted in an expansion of the radiological services and the opportunity to receive a second opinion about images. This is a positive result of the reform and will be monitored once the new equipment and services are all in place.

Whilst acknowledging that stakeholders participating in the study agree that decentralised radiological services in the region are necessary and indeed are a requirement by politicians, professions and citizens, it is the view of the authors based on this study that services could be delivered more efficiently and effectively. Furthermore, a Swedish study found that decentralising health services can lead to local variation or inequality in services. Moreover there is a potential conflict between central and local decision making [1]. Organisational culture emerges from that which is shared between colleagues in an organisation, including shared beliefs, attitudes, values, and norms of behavior [43]. Results from this study show that managers are taking the cooperation issue seriously, but not all problems have been resolved, there are still certain problems like common IT systems and procedures. Ferlie and Shortell [44] state that the lack of integrated IT systems can inhibit a high quality health care system. Although Hillestad, et al. [45] are of the opinion that IT systems have potential for savings in the health care system; however they have to be fit for purpose. Technological development has the potential to be better utilised; digital imaging and tele-radiology have fundamentally changed radiology inasmuch as radiological images can now be sent electronically from a remote location to a radiologist in another location for interpretation or consultation [46]. Therefore, common IT platforms have the potential to facilitate knowledge sharing and ultimately improving *quality*. However, as noted earlier such developments brings with them data protection and security issues.

### Limitations of the study

The current study is based on a single case study aimed to obtain and understand stakeholder experiences and perspectives of one decentralised radiological service in a rural area of Norway, which had developed organically prior to the recent reforms. This qualitative study has certain limitations. Results reported from a limited sample from one area using only two focus groups, may not be transferable to other settings. The study findings can therefore not be broadly generalised to other areas or radiological departments or radiographers, but it does provide some insights for key stakeholders to consider. Other approaches may have provided different perspectives.

Despite this limitations the study assumes importance given the lack of publications on decentralised radiological services. It will augment our understanding of the challenges posed by the change in radiological services at a local level, based on experience from stakeholders using such services in one region in Norway.

For decentralising radiological service, there still seem to be hindrances, such as funding and limited cooperation, to an effective high-quality service. Future research topics could be to identify and explore these hindrances and to identify ways of overcoming them. The safety, data protection and security risks associated with radiological services make it a high priority for further research at national and international levels.

## Conclusion

Experiences from a rural decentralised radiological service reveals challenges in **organisation** of the health service further challenging the national health care system The three-fold overlapping structure could be more effective by merging the radiological service at the LMC with the emergency x-ray unit in the same building. Regarding **quality and safety,** the study has revealed significant human capital deficits given that the radiological services in the GP practicses and in the acute unit at the LMC need to improve the quality of x-ray imaging and the interpretation of images. Since there is non- radiographic staff taking x-rays in the GP practices, this study concludes that training and knowledge transferring in imaging and radiation protection is needed for non-radiographic staff including GPs. Findings in this study indicate that **funding** challenges between the specialist and the primary health services remain unresolved. The health care funding system in Norway where hospitals are funded from the government level, and community health care from every local municipality, seems to hinder equal health services. Lack of **cooperation** between professions and between the specialised and primary care levels. There are concerns about differences in thinking between “the hospital” (fragmented thinking) and “district medicine” (holistic thinking). The study also indicates that responsibility between levels so that patients’ pathways are better managed.

## Supplementary information

**Additional file 1: Table S1.** Interview guide.

## Data Availability

The datasets generated and analysed during the current study are not publicly available due to participant anonymity issues but are available from the corresponding author on reasonable request.

## Abbreviations

GP: General practitioner
IAEA: International Atom Energy Agency
LMC: Local Medical Centre
