# Supplementary material for: Experiences from Decentralised Radiological Services in Norway – a rural case study
Source: BMC Health Serv Res. 2019 Dec 12;19:959. doi: 10.1186/s12913-019-4800-z (PMC6909618; doi:10.1186/s12913-019-4800-z)
Supplement: Supplementary file 1 — Additional file 1: Table S1. Interview guide. [file 12913_2019_4800_MOESM1_ESM.docx]

**Contents - Additional file**

Table A1: Interview guide

Table A1: Interview guide

| Parts | Function | Theme | Questions to participants |
| --- | --- | --- | --- |
| Opening  (1.question, few minutes) | To get everyone to talk early in the discussion. The longer it is before someone says something in a group, the less likely he or she is to say something. Designed to be easy and quickly to answer. Best to ask for facts as opposed to attitudes or opinions. Typically not analysed. Intension is to get people talking and to help people to get comfortable. | Introduction and brief description of the roles | Please, do an introduction about yourself, name and your role. Explore how your role is connected to the decentralized radiological service. |
| Introductory  (1 question, few minutes) | Introduce the topic of the discussion and get people to start thinking about their connection with the topic. Encourage conversation. Usually open-ended questions that allow people to tell about how they see or understand the issue under investigation. Sometimes ask participants to remember back when they first experienced the topic under investigation. | Experiences | Describe shortly your experiences with the radiological service in the area, from your viewpoint. |
| Transition | Move the conversation into the key questions that drive the study. Logical links between introductory questions and key questions. Go into more depth than the introductory question about their experiences. | Pick up experiences from the last section | Follow up questions from the last section |
| Key questions (20 minutes) | Key questions drive the study. Typically two to five questions. Important for the moderator to know the key questions. | Exploring decentralization, quality, profession roles, organization and economic consequences | 1. What is your opinion about decentralized health care?  Benefits/ disadvantages  Drivers/ hinders?  2. Your opinion about quality in the service?  3. Your opinion about the organization of the service?  4.Economi in decentralized health care  5. The role of the professions / team work |
| Ending questions  (1-2 questions, 10 minutes) | Used to determine the final position of participants on critical areas. Allows clarifying å position. Valuable to analyse because it helps to interpret conflicting comments and gives weight to what was said. Sometimes trivial concerns are talked about frequently during the focus group, but frequently does not necessarily reflect importance.  Final question,  Insurance question that no critical aspects have been overlooked. | Summary/consolidation | Your opinion, x-ray imaging in the GPs surgery?  Patients satisfied – why?  Your wishes for a future service?  Something more? |
